# Influence of Surface Passivation on AlN Barrier Stress and Scattering Mechanism in Ultra-thin AlN/GaN Heterostructure Field-Effect Transistors

**DOI:** 10.1186/s11671-016-1591-6

**Published:** 2016-08-23

**Authors:** Y. J. Lv, X. B. Song, Y. G. Wang, Y. L. Fang, Z. H. Feng

**Affiliations:** National Key Laboratory of Application Specific Integrated Circuit (ASIC), Hebei Semiconductor Research Institute, Shijiazhuang, 050051 China

**Keywords:** AlN/GaN, SiN passivation, Electron mobility, Polarization Coulomb field scattering

## Abstract

Ultra-thin AlN/GaN heterostructure field-effect transistors (HFETs) with, and without, SiN passivation were fabricated by the same growth and device processes. Based on the measured DC characteristics, including the capacitance-voltage (*C*-*V*) and output current-voltage (*I*-*V*) curves, the variation of electron mobility with gate bias was found to be quite different for devices with, and without, SiN passivation. Although the AlN barrier layer is ultra thin (*c*. 3 nm), it was proved that SiN passivation induces no additional tensile stress and has no significant influence on the piezoelectric polarization of the AlN layer using Hall and Raman measurements. The SiN passivation was found to affect the surface properties, thereby increasing the electron density of the two-dimensional electron gas (2DEG) under the access region. The higher electron density in the access region after SiN passivation enhanced the electrostatic screening for the non-uniform distributed polarization charges, meaning that the polarization Coulomb field scattering has a weaker effect on the electron drift mobility in AlN/GaN-based devices.

## Background

Attributed to the high critical field and electron velocity, nitride heterostructures have attracted great attention because of the excellent potential application in high-voltage and high-power operations at microwave/sub-microwave frequency [1–5]. Thanks to the large band-gap energy and conduction-band offset to GaN, AlN/GaN heterostructures with ultra-thin barrier layer (~3 nm) are expected to be important in three-dimensional device scaling in order to obtain high frequencies, enabling the realization of millimeter-wave and/or even sub-millimeter-wave power devices [6–8]. Using device-scaling technologies, the HRL Laboratory has reported a D-mode AlN/GaN heterostructure field-effect transistor (HFET) with an ultra-high *f*_T_ exceeding 450 GHz and a *f*_max_ close to 600 GHz, which are the best frequency characteristics yet found in GaN-based HFETs [9]. Nowadays, the surface passivation usually uses an SiN dielectric grown by a plasma enhanced chemical vapor deposition (PECVD) system, which has been demonstrated to be an effective material when mitigating against current collapse in Al_*x*_Ga_1-*x*_N/GaN HFETs, to a certain extent regulating the two-dimensional electron gas (2DEG) density [10–19]. It has been proven that the increase of 2DEG density is not due to the induced stress in the barrier layer but the influence of SiN passivation on the surface properties of the AlGaN barrier layer: the potential barrier height of the AlGaN barrier layer is then changed after SiN passivation, resulting in a 2DEG density change [18–21]. However, in the AlN/GaN heterostructure, the AlN barrier layer is ultra thin (*c*. 3 nm) and has large piezoelectric polarization, so the crystal lattice of the AlN layer may be more sensitive to SiN passivation. Whether the SiN passivation induces additional stress in the ultra-thin AlN barrier layer remains unknown. Moreover, the polarization Coulomb field (PCF) scattering related to the non-uniform distribution of polarization charges in the barrier layer has been demonstrated to be an important mechanism in AlGaN/GaN HFETs [22–25]. The PCF scattering exerts a dominant influence on electron drift mobility in AlN/GaN HFETs due to the thin barrier layer [26, 27]. Whether the SiN passivation induces additional stress in the AlN barrier layer or just affects the surface properties of the AlGaN barrier layer, the increase in 2DEG after SiN passivation will affect the PCF scattering, which influences electron mobility in AlN/GaN HFETs. As a result, it was deemed worthwhile to investigate the influence of SiN passivation on the ultra-thin AlN barrier layer and the transport properties in AlN/GaN HFETs.

In this work, ultra-thin AlN/GaN HFETs with, and without, SiN passivation were fabricated with the same growth and device processes, respectively. Using the measured DC characteristics, including the capacitance-voltage (*C-V*) and output current-voltage (*I-V*) curves, it was found that the electron mobility varied with gate bias quite differently for devices with, and without, SiN passivation. Based on Raman and Hall measurements of the AlN/GaN heterostucture with different SiN thicknesses, SiN passivation was proved to exert no significant influence on the piezoelectric polarization of the AlN barrier layer, but to have affected the surface properties of the AlN/GaN heterostructure. As a result, the increase in electron density in the access region weakened the effect of PCF scattering in ultra-thin AlN/GaN HFETs after SiN passivation.

## Methods

An ultra-thin AlN/GaN heterostucture, from top to sapphire substrate, was formed with a 1-nm GaN cap layer, a 3-nm AlN barrier layer, a 2.5-μm S. I. GaN buffer layer, and a low-temperature AlN nucleation layer, which was grown by metal organic chemical vapor deposition (MOCVD). From room-temperature Hall measurements, the sheet carrier density and electron drift mobility were found to be around 8.92 × 10^12^ cm^−2^ and 1510 cm^2^ V.s^−1^, respectively. The device mesa was isolated by reactive ion etching with Cl_2_/BCl_3_ gas. Ohmic contacts with Si/Ti/Al/Ni/Au metal stacks were deposited by e-beam evaporation and lift-off and then annealed rapidly in a nitrogen atmosphere to form good Ohmic contacts. The specific resistivity was found to be 5.9 × 10^−5^ Ω cm^2^ by transmission line method (TLM). The rectangular Ohmic contacts were 50 μm long and 100 μm wide, with a source-to-drain distance of 100 μm. Through e-beam evaporation and lift-off technology, Schottky contacts with Ni/Au metal stacks were deposited in the center between the drain and source contacts, and the size of each Schottky contact was 20 μm long by 100 μm wide. Finally a 100-nm-thick SiN passivation layer was deposited by PECVD. To compare devices with, and without, SiN passivation, unpassivated AlN/GaN HFETs were also prepared with the same growth and device processes. Besides, classical van der Pauw Hall structures were fabricated on the same wafer during processing. Each pattern was a 500 μm × 500 μm square mesa.

## Results and Discussion

**Fig. 1:**
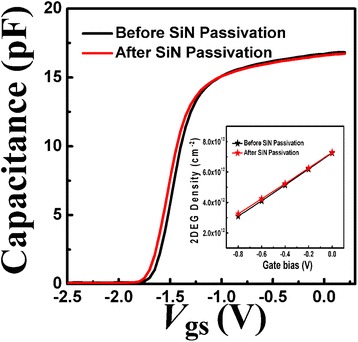
The measured *C-V* curves for the prepared AlN/GaN HFETs before and after SiN passivation

The tested *C-V* curves of the Schottky-to-source contacts for the prepared AlN/GaN HFETs with/without SiN passivation are shown in Fig. 1. During the measurements, an Agilent B1520A system was used and the tests were conducted at a frequency of 1 MHz, at room temperature, using the source and Schottky contact. The gate bias ranged from 0.5 to −2.5 V in increments of −0.05 V. By integrating the tested *C-V* curves, the 2DEG density (*n*_2D_) over a range of gate biases can be extracted [10], and the calculated results are shown in Fig. 1. There may be about ±1 % error between the calculated and authentic 2DEG densities, because of the error between the design and the manufacture of each Schottky contact. It can be seen that the *C-V* curve moved slightly towards a reverse orientation after SiN passivation. The 2DEG densities, at different gate biases, were quasi-constant, despite SiN passivation. This indicated that the SiN passivation exerted no influence on the stress or surface states of the AlN barrier layer underneath the Ni/Au contact due to the presence of Schottky metals.

In rectangular AlN/GaN HFET devices, the 2DEG electron drift mobility under the Schottky contact can be extracted from the measured DC characteristics as follows [26]:

1 $$ {\mu}_n=\frac{I_{DS}{L}_G}{q{n}_{2D}W\left[{V}_{DS}-{I}_{DS}\left({R}_D+{R}_S+{R}_{\mathrm{Ohm}}\right)\right]} $$

2 $$ {R}_D=\frac{L_{GD}}{q{n}_{2D0}{\mu}_{n0}W} $$

3 $$ {R}_S=\frac{L_{GS}}{q{n}_{2D0}{\mu}_{n0}W} $$

where *R*_D_ and *R*_S_ are the gate-to-drain and gate-to-source channel resistances, respectively, *R*_Ohm_ is the total Ohmic resistance of both source and drain contacts, *L*_GD_ and *L*_GS_ are the gate-to-drain and gate-to-source distances, respectively, *L*_G_ is the gate length, *W* is the gate width, and *n*_2D0_ and *μ*_n0_ are the 2DEG electron density and drift mobility in the access region, respectively. Other parameter definitions were taken from the literature [23].

**Fig. 2:**
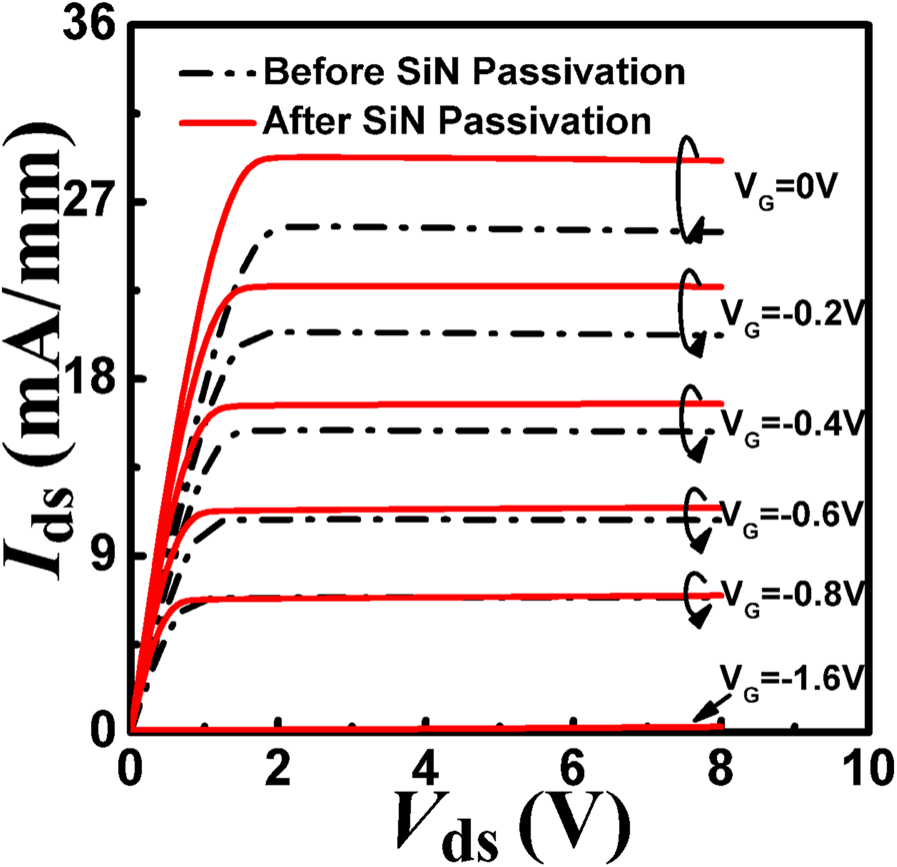
The measured output characteristics for the prepared AlN/GaN HFETs before and after SiN passivation

**Fig. 3:**
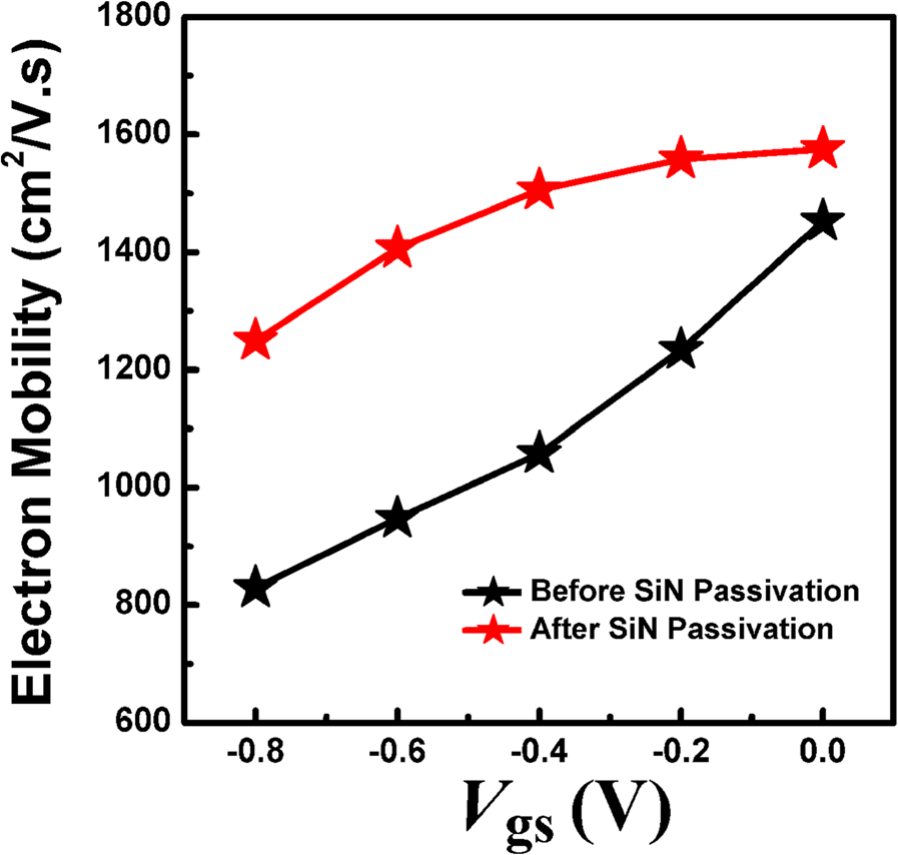
The variation in 2DEG electron mobility underneath the gate area for different gate biases, at room temperature, in the fabricated AlN/GaN HFETs before, and after, SiN passivation

Figure 2 shows the output characteristics of the fabricated AlN/GaN HFETs with/without SiN passivation. During room-temperature measurement, the drain-source voltage ranged from 0 to 8 V in increments of 0.05 V, while the gate bias ranged from 0 to −1.6 V in increments of −0.2 V. The 2DEG electron mobility underneath the gate contact for the ultra-thin AlN/GaN HFETs with/without SiN passivation was calculated using Eq. (1), and the results are shown in Fig. 3. During the calculation, the drain current (*I*_DS_) at 100 mV drain bias with different gate biases was used. Before SiN passivation, the electron mobility under the Schottky contact increased monotonically with increasing forward gate voltage. After SiN passivation, the electron mobility increased at the same gate bias compared to that without SiN passivation. Moreover, the electron mobility increased much more slowly upon increasing the forward gate voltage and almost reached saturation as the gate bias tended to zero. The deviation of electron mobility between gate biases of −0.8 and 0 V became much smaller after SiN passivation, mainly due to the varied effect of the scattering mechanism on the electron mobility. To investigate the scattering mechanism in AlN/GaN HFETs with/without SiN passivation, the influence of SiN passivation on the AlN barrier layer and 2DEG density is discussed as follows.

**Fig. 4:**
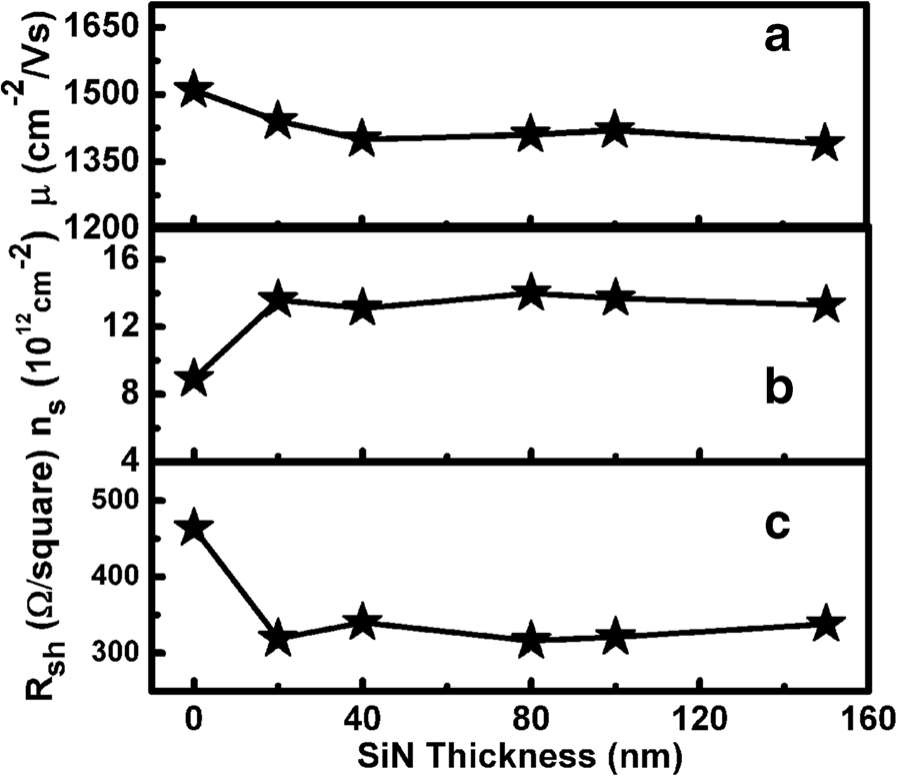
**a** Measured electron mobility (*μ*), **b** 2DEG electron density (*n*_s_), and **c** areal resistance (*R*_sh_) of AlN/GaN heterostucture, at room temperature, as a function of SiN thickness

Figure 4 shows (a) the room-temperature electron mobility, (b) 2DEG electron density, and (c) areal resistance of the AlN/GaN HFETs before, and after, SiN deposition, as a function of SiN thickness. The measurements were based on the fabricated van der Pauw Hall patterns. The thickness of the passivated SiN layer was proved by ellipsometer. After deposition of a 20-nm thickness of SiN, the electron mobility decreased from 1510 to 1440 cm^2^ V.s^−1^. On the other hand, the 2DEG electron density increased, after deposition of a 20-nm thickness of SiN, from 8.92 × 10^12^ to 1.36 × 10^13^ cm^−2^. The areal resistance decreased, after deposition of a 20-nm thickness of SiN, from 464 to 319 Ω V.s^−1^. Moreover, the electron mobility, electron density, and areal resistance did not change upon further increasing the thickness of the SiN passivation layer. If the SiN passivation can induce additional tensile stress in the AlN barrier, the stress should be increased upon increasing the SiN layer thickness. So, it was unlikely that the increasing 2DEG density was caused by enhanced piezoelectric polarization. This demonstrated that the SiN passivation induced no additional tensile stress in the AlN layer and exerted no significant influence on the piezoelectric polarization of the AlN barrier layer. The fact that the 2DEG density increased after SiN passivation was mainly due to the reduction of the surface states and AlN potential barrier height [21]. It is usually thought that the surface states are related to the negative charges fixed at the surface. Since the space between the surface, and the 2DEG electron, is small, the influence of the electric field induced by the surface states on the electron density is much stronger for ultra-thin AlN barriers, and the elimination of surface states is more significant with regard to any improvement in the characteristics of the AlN/GaN heterostructure, meaning that the electron density and sheet resistance changed significantly after even 20 nm of SiN passivation. The reason for the reduction in electron mobility after SiN passivation was possibly that the increased rate of electron–electron and interface roughness scattering caused a decrease in mobility, because higher density carriers were confined to within a smaller region due to the increased electron density.

**Fig. 5:**
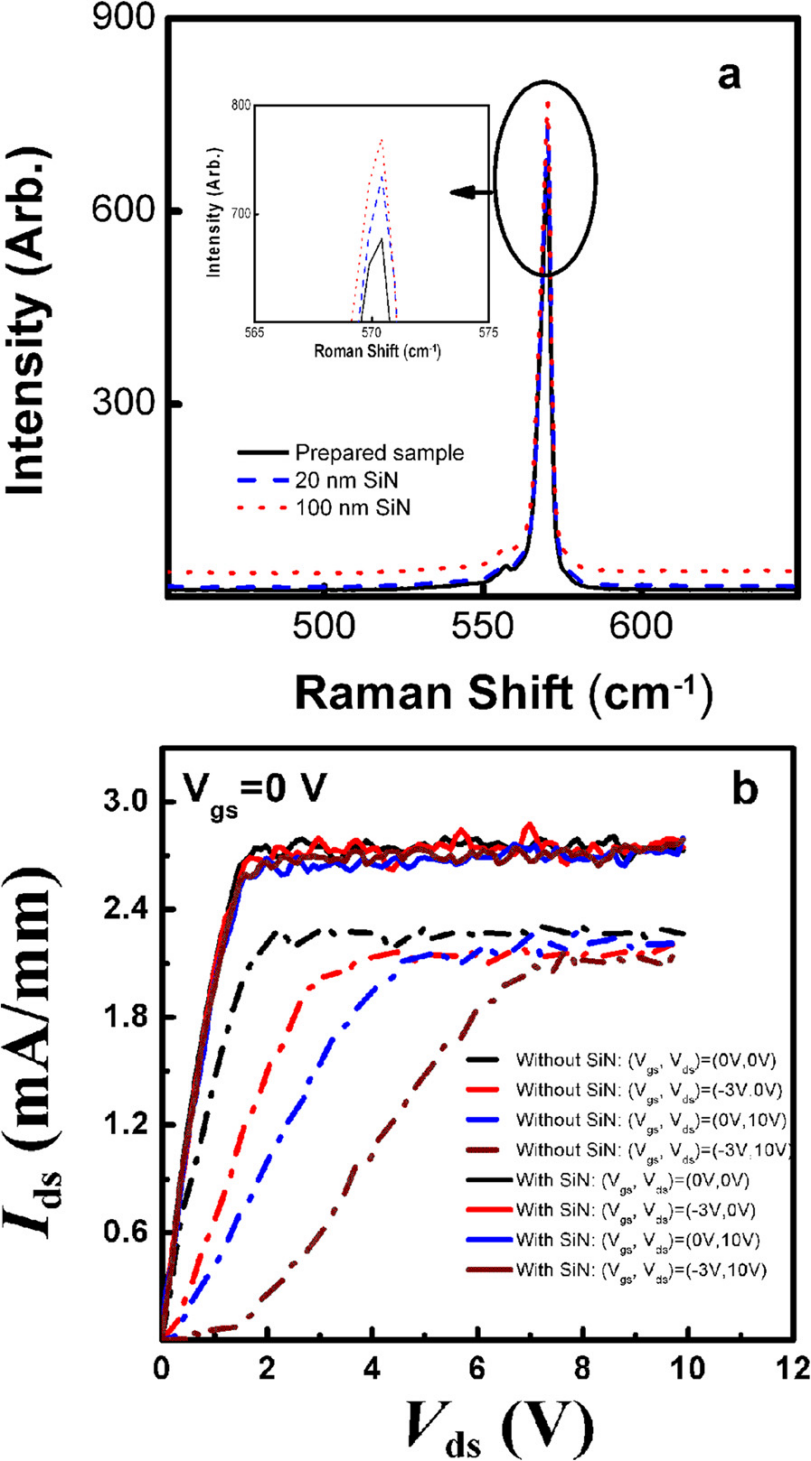
**a** Raman measurements of AlN/GaN heterostucture with 0, 20, and 100 nm SiN passivation. **b** Pulse output at *V*_gs_ = 0 V with different bias points for AlN/GaN HFETs with/without SiN passivation

The Raman measurements of AlN/GaN heterostucture with 0-, 20-, and 100-nm SiN passivation layers are shown in Fig. 5a. The wavenumber of the GaN buffer in the AlN/GaN heterostucture was found to have been 570.4 cm^−1^. When the sample was passivated with 20 or 100 nm SiN, the wavenumber of the GaN buffer underwent no shift either left or right, further indicating that the SiN passivation exerted no significant influence on the piezoelectric polarization of the AlN barrier layer. Besides, the pulse output characteristics under different bias conditions were also measured as shown in Fig. 5b. Before SiN passivation, there was a significant difference in drain current between quiescent points (*V*_gs_, *V*_ds_) of (0, 0) and (−3, 0), and the difference was further increased when the quiescent point (*V*_gs_, *V*_ds_) was set to (0, 10) and (−3, 10), respectively. However, there was almost no drain current dispersion after SiN passivation. This demonstrated that the SiN passivation effectively eliminated the surface states and reduced the trapping effects which induced gate-lag or drain lag [18, 28].

**Fig. 6:**
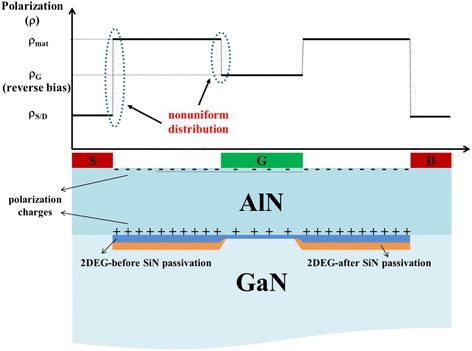
Schematic diagram for the distribution of the polarization charge and 2DEG electron density in the AlN/GaN HFETs

Figure 6 shows the arrangement of the 2DEG and polarization charges for the ultra-thin AlN/GaN HFETs. In Fig. 6, *ρ*_Mat_ is the density of polarization charge of the AlN/GaN heterostructure. The density of the polarization charge underneath the Ni/Au metal stacks is denoted by *ρ*_*G*_, and the density of the polarization charge underneath the source, or drain, electrodes is denoted by *ρ*_*S/D*_. According to previous results, metal atoms spread into the AlN barrier layer due to high-temperature annealing, resulting in the value of *ρ*_*S/D*_ being smaller than that in the AlN/GaN heterostructure [29, 30]. Moreover, the Ni/Au metal may interact with the surface atoms, or states, of the AlN barrier layer and alter the Schottky barrier height, which will cause the value of *ρ*_*G*_ to also be smaller than that in the AlN/GaN heterostructure [26, 27, 29]. In addition, the converse piezoelectric effect will further alter the stress in the AlN barrier layer under reverse gate bias, which further changed the difference between *ρ*_Mat_ and *ρ*_*G*_. As a result, and induced by the non-uniform polarization among *ρ*_Mat_, *ρ*_*G*_, and *ρ*_*S/D*_, an elastic scattering potential on the electron drift mobility was established, which was named PCF scattering here. Since the AlN barrier layer was very thin, it would bear a much larger electric field and meanwhile a stronger converse piezoelectric effect once a reverse gate bias was applied. Therefore, the PCF scattering became the dominant scattering mechanism in the AlN/GaN HFETs and affected the electron drift mobility, resulting in it decreasing monotonically with a decreased 2DEG electron density (Fig. 3).

In the SiN passivated AlN/GaN HFETs, the SiN passivation induced no additional tensile stress in the AlN layer and had no significant enhancing effect on the PCF elastic potential and scattering of the 2DEG electron mobility. However, the 2DEG density under the access region greatly increased after SiN passivation. Once the 2DEG density increased, the electrostatic shielding on the non-uniform region of the polarization charges among *ρ*_Mat_, *ρ*_*G*_, and *ρ*_*S/D*_ was enhanced, which weakened the effect of PCF scattering on electron mobility under the gate area. Thus, as shown in Fig. 3, the deviation of electron mobility between the gate biases of −0.8 and 0 V became much smaller after SiN passivation. Moreover, the increased electron mobility, after SiN passivation, at the same gate bias was also due to the weaker PCF scattering effect.

## Conclusions

Rectangular HFET devices with, and without, SiN passivation were fabricated on ultra-thin AlN/GaN heterostuctures. Based on the measured DC characteristics, the changes in 2DEG electron mobility underneath the Schottky contacts with applied gate voltage for the fabricated AlN/GaN HFET devices was obtained. Based on Hall measurements of AlN/GaN heterostuctures with different SiN thicknesses, the electron mobility, electron density, and sheet resistance were found to have remained quasi-constant with increasing SiN thickness, which demonstrated that the stress induced by the presence of the SiN film should not be an essential reason for the increased 2DEG density. No wavenumber shift in the GaN buffer further indicated that the SiN passivation exerted no significant influence on the piezoelectric polarization of the AlN barrier layer. Pulse output characteristics further demonstrated that the increased 2DEG density was mainly caused by the reduction of surface states after SiN passivation. The higher electron density in the access region after SiN passivation enhanced the electrostatic screening for the non-uniformly distributed polarization charges, meaning that PCF scattering had a weaker effect on the electron mobility in the AlN/GaN HFETs after SiN passivation.
